# Systematic spatial distortion of quantitative estimates

**DOI:** 10.1007/s00426-020-01390-5

**Published:** 2020-07-16

**Authors:** Samuel Shaki, Martin H. Fischer

**Affiliations:** 1 Ariel University, Ariel, Israel; 2 Department of Psychology, University of Potsdam, Karl-Liebknecht-Strasse 24-25 House 14, 14476 Potsdam OT Golm, Germany

## Abstract

Magnitude estimation has been studied since the beginnings of scientific psychology and constitutes a fundamental aspect of human behavior. Yet, it has apparently never been noticed that estimates depend on the spatial arrangement used. We tested 167 adults in three experiments to show that the spatial layout of stimuli and responses systematically distorts number estimation, length production, and weight reproduction performance. The direction of distortion depends on the observer’s counting habits, but does not seem to reflect the use of spatially associated number concepts. Our results imply that all quantitative estimates are contaminated by a “spell of space” whenever stimuli or responses are spatially distributed.

## Introduction

Our behavior includes and requires constant assessments of magnitudes around us: how we estimate the number and weight of objects, as well as distances and durations, determines our actions. The scientific study of such estimations aims “… to control the observer’s […] use of numbers in magnitude estimation such as line-length scaling, magnitude matching, master scaling and category-ratio scaling” (Gescheider, 1997, p. x). While the accuracy of such psychophysical estimations has been thoroughly documented ever since Fechner (1860), the effects of the spatial layout on perceptual performance (expressed by producing numbers or magnitudes) have been largely ignored (e.g., Parth & Rentschler, 1984).

One of the few exceptions to this general state of the literature is the work of Polzella, DaPolito, and Hinsman (1977) who reported that the duration of dot patterns flashed to the left visual field is perceived to be shorter than the duration of the same stimuli flashed to the right visual field. This is an early report of a profound association between perceptual quantity and space. It was revealed by asking participants to enumerate the dots corresponding to the perceived durations. Relatedly, Styrkowiec, Jurczyk, and Lerpec (2020) recently instructed participants to perform head turns to their left side or right side, and showed that environmental distances on one’s right side were overestimated.

Here, we ask: (a) whether such use of numbers to express estimates about spatially distributed stimuli contaminates performance; (b) whether estimation performance depends on the spatial relationship between stimulus, observer, and response; and (c) whether idiosyncratic motor habits of our observers further modulate their psychophysical performance signatures. We begin by motivating these inter-related question from a brief review of findings pertaining to the now well-established association between numbers and space.

Most Western adults associate left space with small numbers and right space with larger numbers. This spatial–numerical association (SNA) has been extensively documented in speeded number classification tasks with lateralized button responses, now widely known as SNARC effect (for *spatial*–*numerical association of respon*se *codes*; Dehaene, Bossini, & Giraux, 1993; for reviews, see Fischer & Shaki, 2014; Knops, 2020): Responses are faster and more accurate for small numbers with left-side responses and for larger numbers with right-side responses. Recently, SNAs were also documented in newborn humans (de Hevia, Veggiotti, Streri, & Bonn, 2017; Di Giorgio, Lunghi, Rugani, Regolin, Dalla Barba, Vallortigara, & Simion, 2019) and chicks (Rugani, Vallortigara, Priftis, & Regolin, 2015) with the use of non-symbolic visual quantities, pointing to a fundamental and pre-conceptual link (Felisatti, Laubrock, Shaki, & Fischer, 2020a, 2020b). Whether such behavioral effects on speed and accuracy also lead to biased quantity appreciations is unknown and requires contrasts against veridical performance, as we will show below.

Importantly, SNAs exist at the perceptual, motorical, and conceptual level: lateralized visual stimulation influences the processing of centrally presented small or large numbers (e.g., Stoianov, Kramer, Umilta, & Zorzi, 2008). In turn, centrally presenting small or large numbers can improve visual perception on one’s left side or right side, respectively (e.g., Fischer, Castel, Dodd, & Pratt, 2003; Casarotti, Michielin, Zorzi, & Umilta, 2007; but see Pellegrino et al., 2019, and Colling et al., 2020). The presence of motorical SNAs was shown by congruency effects in a pointing task (Fischer, 2003), effects of head turning on random number generation (e.g., Loetscher, Schwarz, Schubiger, & Brugger, 2008), and bi-directional associations between walking direction and number magnitude in a random number generation task (Shaki & Fischer, 2014). Finally, purely conceptual SNAs have been documented without contamination from either encoding- or response-related spatial biases (for methodological details, see Fischer & Shaki, 2016; Shaki & Fischer, 2018a). Interestingly, culturally acquired directional reading and counting habits can modulate or even reverse the direction of SNAs (Fischer, 2008; Shaki, Fischer, & Petrusic, 2009; Shaki & Fischer, 2017).

Taking into consideration these known facts about the ubiquitous SNAs, we aimed to address our three questions listed above. Table 1 shows how we progressively identified our answers. In the first experiment, we studied the perceptual-to-conceptual mapping of quantities by asking participants to map a lateralized non-symbolic visual quantity onto a number name. This established the expected influence of stimulus locations on estimations in a typical psychophysical procedure. Moreover, we assessed the spatial direction of object counting habits of our participants (left-to-right vs. right-to-left), because we previously documented that this culturally transmitted preference modulates SNAs (Fischer & Shaki, 2017). Preferred counting direction seems to be less consistent in Hebrew speakers compared to adults from other cultures, presumably due to opposing scanning directions for text vs. numbers (Shaki, Fischer & Göbel, 2012). We hypothesized that the starting side of counting would establish a general association of that side with smaller quantities.

**Table 1:** Overview of experimental tasks and conditions. Cell entries “yes” and “no” indicate whether the experimental condition includes this potential source of distortion of participants’ quantitative estimates

Experiment	*N* (counting direction)	Stimuli	Lateralized encoding	Lateralized response	Number concepts
1. Naming numerosity	21 (left–right) 13 (right-left)	Visual dots	Yes	Yes	Yes
2. Length production	16 (left–right) 13 (right-left)	Digits	No	No	Yes
		(a)			
		(b)	Yes	Yes	
		(c)	No	Yes	
		(d)	Yes	No	
3. Weight reproduction	60 (left–right) 44 (right-left)	Weights	No	Yes	No

In Experiment 2, we required participants to perform the inverse mapping by translating a conceptual symbolic number onto a perceptual quantity. Here, we manipulated the spatial locations of both stimuli and responses to dissociate contributions of lateralized encoding and lateralized responding to overall estimation performance. Finally, in Experiment 3, we examined a non-visual modality and removed conceptual number processing from both stimuli and responses to show that estimation performance is distorted by spatial manipulations even in the absence of number names.

## General method

We begin with a description of common methodological aspects of all experiments. All participants were native Hebrew readers with self-reported normal or corrected-to-normal vision and were naïve about the purpose of the experiments. They participated for course credit after signing an informed consent form. The experiments were approved by the University’s Ethics Review Board and carried out in accordance with the provisions of the World Medical Association’s Declaration of Helsinki. Sample sizes were based on our previous experience with counting-habit-related effects (Fischer & Shaki, 2016, 2017), except for the third experiment where the reduced number of trials per participant was compensated by a substantial increase in sample size to retain sufficient statistical power (Baker et al., 2019). Data were collected by an undergraduate RA and we used SPSS software (versions 25 and 26) for all analyses.

At the end of each experiment, we administered a previously introduced test (Fischer & Shaki, 2017) to assess the direction of SNAs: Each participant sat down at a table across from the experimenter. Four identical black cardboard circles with a diameter of 4 cm and no writing on them were presented in a linear array on a blank A4-sized piece of paper in landscape format, centered in front of the participant. The circles were placed equidistantly from each other in a fronto-parallel plane. The participant was asked to “count these circles aloud and touch each circle while counting”. No demonstration was given and the participant’s order of counting was recorded in a single trial by the experimenter as either left-to-right or right-to-left.

For each experiment, the participants’ estimates (of numerosity, length, or weight) were analyzed with mixed-factor analyses of variance (ANOVA) and followed up with non-directional *t* tests to document spatially distorted quantity estimations, defined as differences between left side and right side. We also evaluated the presence and direction of systematic biases, defined as differences between lateralized presentations and correct magnitudes, by testing participants’ estimates against the correct magnitudes with non-directional *t* tests. As part of open practices, the raw data and analysis scripts are available at https://osf.io/3mp8w/. However, the study was not pre-registered.

## Experiment 1: numerosity naming

In Experiment 1, with both spatial–numerical stimuli and spatial–numerical responses, participants named the numerosities of dot patterns appearing for 1 s on a screen located either on their left side or right side in separate conditions. The goal of this lateralized estimation task was to establish the influence of stimulus locations on quantitative estimates in a typical psychophysical procedure before further analyzing it.

### Participants

Thirty-five adults (22 females, mean age 23.2 years, range 18–32 years, 4 left-handed) participated in one 10-min session.

### Stimuli and apparatus

Eight dot clouds (comprising 20, 22, 25, 27, 30, 32, 35, and 37 dots) were equally often used as stimuli in all conditions. Dot diameter was six pixels (each pixel measured 0.25 mm): they were randomly distributed inside an invisible circle (diameter = 6 cm) by an in-house program.^1^ Resulting dot clouds were shown in black on white background on a 19-inch display with 1280 × 1024 pixel resolution (landscape orientation), located on either the participant’s left side or right side, while the keyboard was always straight ahead. Presentation of stimuli was software-controlled and responses were made verbally; the experimenter noted these estimates after each trial.

### Design and procedure

Participants were randomly allocated to one of two conditions: left displays first or right displays first. In each condition, participants looked straight ahead and initiated each trial by pressing the space bar on the keyboard in front of them. One second later, the dot cloud appeared on the lateral display for 800 ms, and participants’ task was to estimate how many dots were presented. Participants were instructed to turn their heads toward the lateral screen immediately after they pressed the space bar and to state their estimate before looking again straight ahead. It was not possible to rotate the chair, and thus, there were only minimal upper body rotations.

Each dot cloud was repeated once per condition, resulting in 16 experimental trials for each participant. Moreover, each block of 16 trials was preceded by presentation of two randomly chosen dot clouds for practice. Stimuli order was randomized within condition.

### Results

**Fig. 1:**
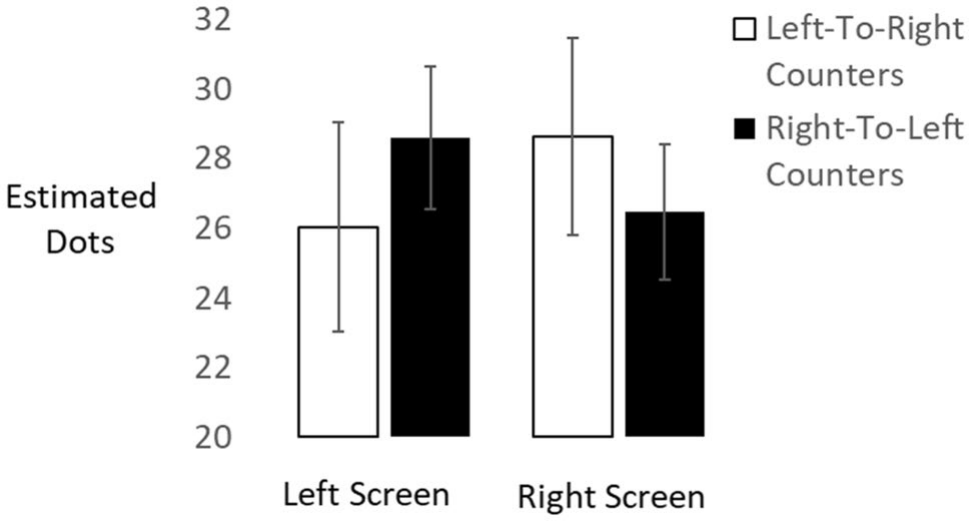
Performance biases observed in the numerosity naming task. Error bars reflect 1 Standard Error of the Mean

Data from one participant were discarded (due to mismatch between the number of total trials and the number of recorded verbal responses) and the remaining 34 data sets were analyzed with a mixed-factors ANOVA to evaluate the effects of screen location (left, right; within participants) and counting preference (left-to-right, right-to-left; between participants) on verbal numerosity estimates. There was a reliable interaction of screen location with counting preference, *F*(1,32) = 6.19, *p* = 0.018, partial *η*^2^ = 0.162. Figure 1 shows numerosity estimates on left and right screens separately for the two groups which differed in the direction of their counting preferences. The 21 left-to-right counters gave marginally smaller estimates when dot clouds were presented on the left (*M* = 26.036 dots, SE = 1.170) compared to the right screen (*M *= 28.619 dots, SE = 1.103), *t*(20) = − 1.85, *p* = 0.079, Cohen’s *d* = 0.44. The 13 right-to-left counters gave instead larger estimates when dot clouds were presented on the left (*M* = 28.596 dots, SE = 1.487) compared to the right screen (*M* = 26.452 dots, SE = 1.402), *t*(12) = 2.59, *p* = 0.024, Cohen’s *d* = 0.53.

When testing these four means against the correct average numerosity of 28.5, we obtained reliable underestimation for left-to-right counters on the left side, *t*(20) = 1.89, *p* = 0.04, but no reliable overestimation on the right side, *t*(20) = 0.97, *p* = 0.41. For right-to-left counters, the pattern was reversed, with reliable underestimation on the right side, *t*(12) = 1.90, *p* = 0.04, and no reliable overestimation on the left side, *t*(12) = 0.08, *p* = 0.47.

Before discussing these findings, we wanted to replicate our results. We conducted a second experiment to understand how lateralized encoding and lateralized responding contribute to the bias (see Table 1). We also aimed to clarify the role of number concepts and removed number names and associated SNAs from stimulus encoding in a third experiment.

## Experiment 2: length production

Perceived length of centrally presented visual intervals is systematically distorted by flanker numbers (e.g., Fischer, 2001; de Hevia et al., 2008). This indicates a contamination of length estimates from number concepts. Given our interest in the role of lateralization on estimation in such tasks, in Experiment 2, we manipulated the spatial location of stimuli and responses in a length production task. Stimuli were symbolic numbers, but responses were non-numerical line lengths to reduce conceptual bias from responding with number words. We compared performance across four conditions differing with regard to stimulus and response lateralization (see Table 1). Importantly, we introduced a baseline condition to compare spatially contaminated estimates against uncontaminated estimates rather than against accurate performance.

### Participants

Thirty-two new students (24 females, mean age 22.8 years, range 21–27 years, 6 left-handed) participated in two sessions (of 25 and 30 min).

### Stimuli and apparatus

**Fig. 2:**
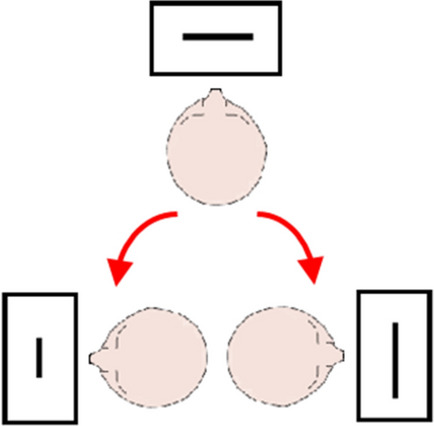
Schematic of the experimental setup, viewed from above. Arrows indicate the head turns of participants in conditions with centrally presented stimuli and lateralized responses while their seat remained fixed. For further details, see main text

Four Arabic digits (3, 4, 6, and 7) were equally often used as stimuli. Digits were shown in black on white background with a Times New Roman font (bold, size = 30 points). Horizontal lines were three pixels thick (each pixel measured 0.25 mm) and appeared in black on white background on the display. As shown in Fig. 2, three identical screens and keyboards were used in the experiment, each at 50 cm viewing distance: one set in front of the participant and the other two to the left and right sides, so participants made approximately 90° head turns to work on the lateralized computers. As before, it was not possible to rotate the chair, and thus, there were only minimal upper body rotations. Presentation of instructions and stimuli, event timing, and response recording were under the control of in-house software. Responses were made with a standard keyboard placed flat on the table with response keys centered under each display.

### Design and procedure

We used a variant of the magnitude production method (Stevens, 1951) developed by Shaki and colleagues (Shaki & Fischer, 2017; Shaki, Sery & Fischer, 2014; Shaki, Pinhas & Fischer, 2018) which removes horizontal spatial biases from participants’ responses. At the beginning of the experiment, two standards, “one unit” (100 pixels) and “ten units” (1000 pixels), were presented on white A4-sized paper (8.3 × 11.7 inches) in landscape orientation. Participants’ task was to produce line lengths matching the magnitudes of the presented digits based on these standards. Participants produced these lines with the “↑” and “↓” arrow keys in four conditions (see Table 1): (a) presentation and production on the same centrally placed display, which served as a baseline; (b) presentation and production on the same lateralized display, either on the left side or right side; (c) central presentation and lateralized production; and (d) lateralized presentation and central production. In the first of two sessions, baseline (a) was always measured before a counterbalanced ordering of sub-conditions (b). In the second session, conditions (c) and (d) were tested, with sub-conditions again counterbalanced in each block.

In each condition, participants were first instructed which displays were relevant in the specific experimental condition, and, accordingly, which keyboards to use for initiating trials and producing the length. Then, the standards were presented for 5 s and participants performed the task without standards being visible. Each of the four digits was repeated seven times per block, which resulted in a total of 196 trials across 7 blocks for each participant. In addition, each block was preceded by one practice presentation and production of each digit. Digit order was randomized within each block.

On each trial, one randomly selected digit was presented 200 ms after the participant pressed an arrow key. The digit was shown for 600 ms; then, the display turned blank until the participant pressed one of the mid-sagittally aligned (near or far) arrow keys to display the starting point of a short line (a ‘dot’, 2 pixels wide) for responding. Each subsequent button press adjusted line length by two pixels (↑ for longer lines, ↓ for shorter lines); continuous pressing adjusted line length at a rate of 30 Hz. Both increasing and decreasing length adjustments were permitted. When participants were satisfied they pressed the “Enter” key to register their response; this started the next trial without feedback.

### Results

Data from three participants (two did not show up to the second session, one had more than 10% missing responses) were discarded and data from the remaining 29 participants were analyzed.

**Fig. 3:**
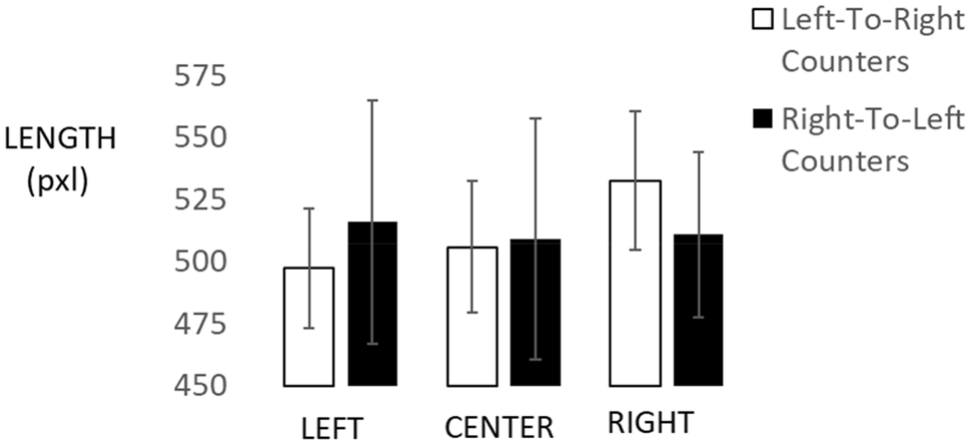
Line length productions when stimuli and responses were in identical locations. Error bars reflect 1 standard error of the mean

Consider first the conditions (a) and (b) with stimuli and responses in identical locations, where condition (a) refers to centrally presented stimuli and responses, while condition (b) refers to lateralized stimuli and responses. Figure 3 shows the results obtained with a mixed-factors ANOVA that evaluated effects of stimulus/response location (left, center, right; within participants) and counting direction (left-to-right, right-to-left; between participants) on length productions. We found a significant interaction of condition with counting direction, *F*(2, 54) = 3.77, *p* = 0.029, partial *η2* = 0.123. Specifically, the 16 left-to-right counters produced shorter lines (*M *= 497.344 pixels, SE = 18.607) on the left side, medium lines (*M *= 505.884 pixels, SE = 18.926) in the center, and longest lines on the right side (*M *= 532.513 pixels, SE = 15.161). Statistical contrasts were: *t*(15) = 1.2, *p* = 0.243, for left vs. center; *t*(15) = 3.16, *p* = 0.007, Cohen’s *d* = 0.49, for right vs. center; and *t*(15) = 3.67, *p* = 0.002, Cohen’s *d* = 0.68, for left vs. right, respectively. Instead, the 13 right-to-left counters produced slightly shorter lines in both central and right conditions (*M *= 509.130 and *M *= 510.907 pixels, respectively, with SE = 20.997 and SE = 16.820), compared to the left condition (*M *= 515.717 pixels, SE = 20.642). However, due to larger variability in this group (see Fig. 3), there were no reliable contrasts, all *t*(12) < 1.

When testing these four means against the correct average length of 500 pixels, we obtained reliable overestimation for left-to-right counters on the right side, *t*(15) = 2.34, *p* = 0.03, but no absolute bias on the center, *t*(15) = 0.44, *p* = 0.66, or the left side, t(15) = − 0.22, p = 0.83. For right-to-left counters, we obtained no absolute bias overall, with *t*(12) = 0.9, *p* = 0.56 for the right side, with *t*(12) = 0.34, *p* = 0.74 for the center, and with *t*(12) = 0.58, *p* = 0.57 for the left side.

**Fig. 4:**
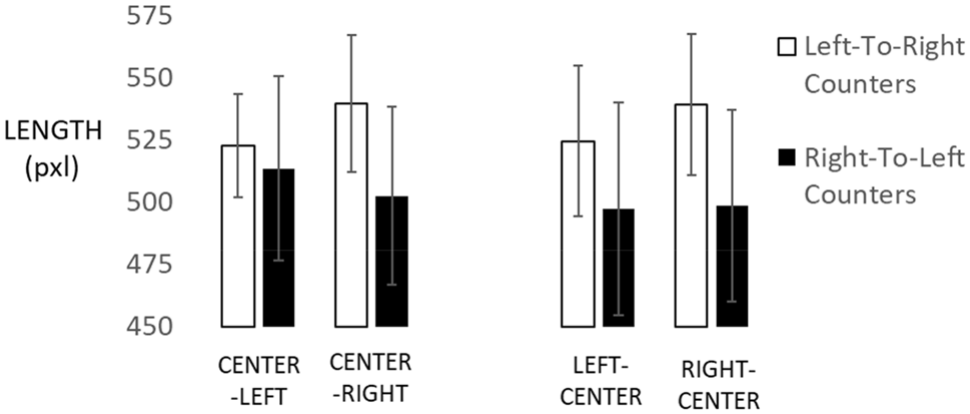
Line length productions when stimuli and responses were in different locations. X-axis labels state stimulus location before response location. Error bars reflect 1 standard error of the Mean

Now, we turn to condition (c) with centrally presented stimuli and lateralized responses. The left side of Fig. 4 shows the results obtained with a mixed-factors ANOVA that evaluated effects of stimulus/response location (center/left, center/right; within participants) and counting direction (left-to-right, right-to-left; between participants) on length productions. The interaction of side with counting direction was again statistically reliable, *F*(1, 27) = 6.14, *p* = 0.020, partial *η*^2^ = 0.185 (see Fig. 4). Specifically, left-to-right counters produced significantly shorter lines on their left compared to their right side (*M* = 522.731 vs. 539.617 pixels, respectively, with S*E* = 14.609 and 15.803), *t*(15) = 2.57, *p* = 0.021, Cohen’s *d* = 0.35. Instead, right-to-left counters produced only numerically longer lines on their left compared to their right side (*M* = 513.523 vs. 502.505 pixels, respectively, with SE= 16.207 and 17.532), *t*(12) = 1.15, *p* = 0.271.

Next, we contrasted individual length estimates against performances in the baseline condition where both stimuli and responses occurred centrally, to assess the contribution of lateralized responding to overall estimates. Only the left-to-right counting group showed a statistically reliable difference: Right-side responses were 34 pixels longer than central responses, *t*(15) = 2,69, *p* = 0.034, all other *p* > 0.34.

When testing these four means against the correct average length of 500 pixels, we obtained reliable overestimation for left-to-right counters on the left side, *t*(15) = 2.19, *p* = 0.04, as well as for the right side, *t*(15) = 2.86, *p* = 0.01. For right-to-left counters, we obtained no absolute bias overall, with *t*(12) = 0.66, *p* = 0.52 for the left side and with *t*(12) = 0.13, *p* = 0.90 for the right side.

Finally, in condition (d) where stimuli were lateralized but responses were given on the central screen, we conducted another mixed-factors ANOVA that evaluated effects of stimulus/response location (center/left, center/right; within participants) and counting direction (left-to-right, right-to-left; between participants) on length productions. As seen on the right side of Fig. 4, we found no reliable interaction of condition with counting direction, *F*(1, 27) = 1.77, *p* = 0.194, partial *η*^2^ = 0.062. Descriptively, left-to-right counters reproduced shorter lines after seeing numbers on their left side compared to their right side (*M *=524.561 vs. 539.144 pixels, respectively, with SE = 18.218 and 16.633), *t*(15) = 1.83, *p* = 0.088, while right-to-left counters produced similar line lengths in the left and right conditions (*M *=497.335 and 498.581 pixels, respectively, with SE = 20.211 and 18.452), *t*(12) < 1.

Next, we again contrasted individual length estimates against performances in the baseline condition where both stimuli and responses occurred centrally, this time to assess the contribution of lateralized stimulation to overall estimates. There were no reliable effects, all *p* > 0.096.

This last analysis indicated that the estimation of spatially distributed quantities was not substantially affected by a further bias. Before discussing our main results, we wanted to document the generality of spatial distortion of quantitative estimations in another modality and without any use of number concepts.

When testing these four means against the correct average length of 500 pixels, we obtained no reliable absolute bias for left-to-right counters on the left side, *t*(15) = 1.62, *p* = 0.13, but a reliable overestimation bias for the right side, *t*(15) = 2.77, *p* = 0.01. For right-to-left counters, we obtained no absolute bias overall, with *t*(12) = − 0.11, *p* = 0.91 for the left side and *t*(12) = − 0.7, *p* = 0.95 for the right side.

## Experiment 3: weight reproduction

In close analogy with the SNARC effect, heavier weights are associated with right space (Dalmaso & Vicovaro, 2019). In Experiment 3, we, therefore, used both non-numerical stimuli and non-numerical responses to replicate our finding with a weight reproduction task. This final experiment served merely to generalize the basic finding of spatially distorted estimations to another modality. For the sake of efficiency and in light of the results from Experiment 2, it contained no baseline to further evaluate possible estimation bias.

### Participants

104 new students (76 females, mean age 22.8 years, range 18–33 years, 14 left-handed) participated in a single 10-min session; the larger sample size compensated for loss of statistical power from fewer trial repetitions (Baker et al., 2019).

### Stimuli and apparatus

Five same-size coffee bowls were prepared as weights. Two bowls were empty and three bowls (tops covered with lids) were partially filled with different amounts of rice (120, 170, and 226 grams total). Two large open containers (diameter 350 mm; height 110 mm) were filled with rice (2000 g) and were located to the participants’ left and right sides, so participants had to make approximately 90^°^ turns with their upper body to fill the empty bowls.

### Design and procedure

Each participant sat across from the experimenter at a small table. In each trial, the participant received one of the three reference ‘weight’ bowls from the experimenter. Participants’ task was to appreciate its weight, set it down, and then produce the exact same weight by filling one of the empty bowls with rice from the left or right source container. The experimenter indicated which source container to use by pointing to it and did not mention the words ‘left’ or ‘right’. Participants were instructed to use their dominant-hand only along the whole experiment. The experimenter then weighed the participant’s bowl on a precision scale, noted the result, and returned the rice to its source container after each trial.

Each of the three reference bowls was randomly given to the participants twice (once to be filled from the left source container and once from the right source container), resulting in six trials for each participant.

### Results

**Fig. 5:**
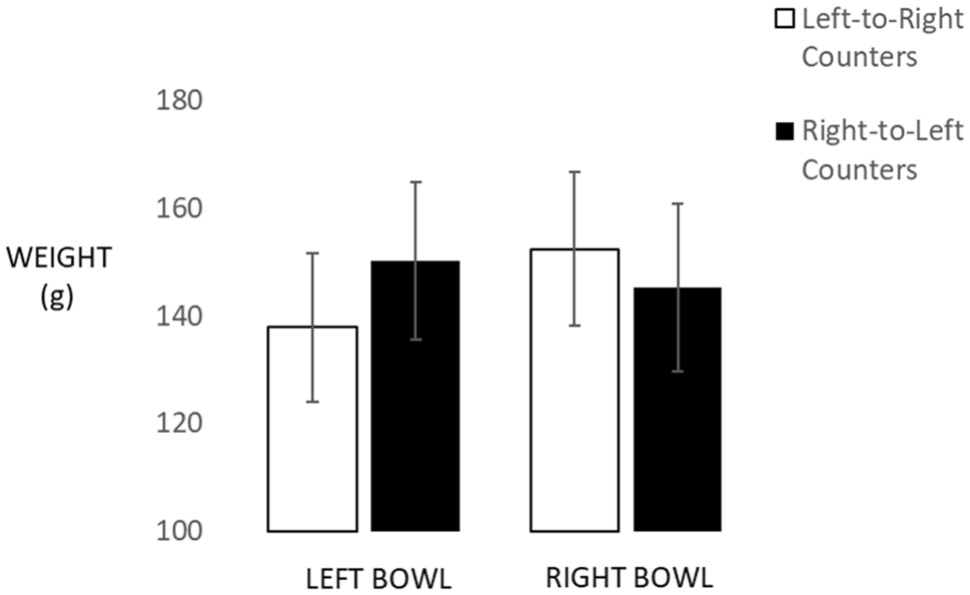
Results of the weight reproduction task. Error bars reflect 1 standard error of the Mean

Figure 5 shows the results obtained with a mixed-factors ANOVA that evaluated the effects of bowl location (left, right; within participants) and counting direction (left-to-right, right-to-left; between participants) on weight reproductions. We found a reliable interaction of bowl location with counting direction, *F*(1, 102) = 58.10, *p* < 0.001, partial *η*^2^ = 0.36. Figure 5 shows that our 60 left-to-right counters produced lighter weights with the left compared to the right bowl (*M *=137.85 g vs. 152.467 g, respectively, with SE = 43.136 and 3.211), *t*(59) = 8.79, *p* = 0.001, Cohen’s *d* = 0.52. In contrast, the 44 right-to-left counters produced heavier weights with the left than the right bowl (*M *=150.212 vs. 145.318 g, respectively, with SE = 3.662 and 3.750), *t*(43) = 2.51, *p* = 0.016, Cohen’s *d* = 0.16.

When testing these three means against the correct average weight of 172 g, we obtained reliable underestimation for left-to-right counters on the left side, *t*(59) = − 11.00, *p* = 0.0001, and on the right side, *t*(59) = − 6.75, *p* = 0.001. Similar absolute bias was obtained for right-to-left counters, with reliable underestimation on the left side, *t*(43) = − 5.88, *p* = 0.001, and also on the right side, *t*(43) = − 6.35, *p* = 0.001.

## Discussion

Three experiments revealed, for the first time, that all magnitude estimations reflect, in addition to our sensory capacities, the spatial aspects of their recording. This is true even when no explicit number concepts are involved. These results identify a previously overlooked fundamental constraint of our appreciation of the world: lateral positioning of stimuli or responses induces a pervasive spatially induced distortion of quantitative estimations. We discuss the evidence obtained with three different methods in turn.

The numerosity naming task established that the spatial layout of the assessment distorts magnitude estimation and that this estimate is also sensitive to counting preferences. Moreover, we obtained these results with a frequently used procedure that occurs in everyday situations as well as in psychophysical assessments, thus highlighting the theoretical and practical relevance of our finding. However, our procedure relied on both spatial–numerical stimuli and numerical responses. This left open the possibility that the distortion was either induced through lateralized number encoding or during indirect SNA activation from number-based responding. We, therefore, removed explicit use of number concepts from responding in a length production task, but still found distorted quantity estimates. Moreover, this experiment dissociated contributions from stimulus encoding and responding to overall bias. Finding significant differences only for lateralized responding and not for lateralized stimulation points to a response-related origin of such bias. Finally, to remove number concepts and associated SNAs also from stimulus encoding, we examined a non-numerical weight reproduction task, and confirmed that even without explicit number concepts, the distortion of quantitative estimates prevailed.

Counting habits imposed a strong influence on all estimations: numerosities were estimated smaller on the side from which participants habitually start to count. The same distortion was again evident in length productions without numerical responses and also influenced weight estimates, even though numbers were never part of the task. This suggests that distorted quantitative estimates are not caused by explicitly activating conceptual number knowledge.

Although our results were obtained with educated human adults, we eventually removed number knowledge from the procedure (cf. weight reproduction task) and thereby made our procedure comparable to SNA demonstrations pointing to evolutionary origins. Specifically, we may have tapped into a pre-conceptual mapping between magnitudes and space that also exists in human newborns (de Hevia et al., 2017; Di Giorgio, Di Giorgio et al., 2019) and some animals (Rugani et al., 2015). We believe that this evolutionarily inherited association reflects neuronal tuning of the brain hemispheres (Felisatti, Laubrock, Shaki, & Fischer 2020a, b) that is subsequently modulated by culturally acquired directional habits, such as reading or finger counting (Shaki & Fischer, 2018b). An interesting observation is that right-to-left counters seem to have weaker SNAs—a finding reminiscent of the modulatory effect of finger counting on SNARC in adults (Fischer, 2008) and awaiting further investigation.

In conclusion, even when we take numbers out of the equation, the relative position of an object influences how we perceive its quantitative physical dimensions. The present findings were obtained in adults with bi-directional (Hebrew and English) reading habits. Future research should examine whether and from what age onwards reading direction habits modulate our distorted quantitative estimates and also establish its reference frame(s).

## Data Availability

The datasets generated and analyzed during the current study are available in the Open Science Framework repository at https://osf.io/3mp8w/.

## Footnotes

1 For our purpose, it was not necessary to control area, density, or other co-varying perceptual dimensions of the dot clouds (e.g., Gebuis et al., 2016; De Marco & Cutini, 2020).
